# Luteal Coasting and Individualization of Human Chorionic Gonadotropin Dose after Gonadotropin-Releasing Hormone Agonist Triggering for Final Oocyte Maturation—A Retrospective Proof-of-Concept Study

**DOI:** 10.3389/fendo.2018.00033

**Published:** 2018-02-15

**Authors:** Barbara Lawrenz, Suzan Samir, Nicolas Garrido, Laura Melado, Nils Engelmann, Human M. Fatemi

**Affiliations:** ^1^IVF Department, IVI Middle-East Fertility Clinic, Abu Dhabi, United Arab Emirates; ^2^Obstetrical Department, Women’s University Hospital Tuebingen, Tuebingen, Germany; ^3^Statistical Department, IVI Foundation, Valencia, Spain

**Keywords:** luteal coasting, GnRH agonist trigger, ovarian hyperstimulation syndrome, individualization of human chorionic gonadotropin dosage, luteal phase support

## Abstract

Ovarian stimulation in a gonadotropin-releasing hormone (GnRH) antagonist protocol with the use of GnRH agonist for final oocyte maturation is the state-of-the-art treatment in patients with an expected or known high response to avoid or at least reduce significantly the risk for development of ovarian hyperstimulation syndrome (OHSS). Due to a shortened LH surge after administration of GnRH agonist in most patients, the luteal phase will be characterized by luteolysis and luteal phase insufficiency. Maintaining a sufficient luteal phase is crucial for achievement of a pregnancy; however, the optimal approach is still under debate. Administration of human chorionic gonadotropin (hCG) within 72 h rescues the corpora lutea function; however, the so far often used 1,500 IU still bear the risk for development of OHSS. The recently introduced concept of “luteal coasting” individualizes the luteal phase support by monitoring the progesterone concentrations and administering a rescue dosage of hCG when progesterone concentrations drop significantly. This retrospective proof-of-concept study explored the correlation between hCG dosages ranging from 375 up to 1,500 IU and the progesterone levels in the early and mid-luteal phases as well as the likelihood of pregnancy, both early and ongoing. The chance of pregnancy is highest with progesterone level ≥13 ng/ml at 48 h postoocyte retrieval. Among the small sample size of 52 women studied, it appears that appropriate progesterone levels can be achieved with hCG dosages as low as 375 IU. This may well optimize the chance of pregnancy while reducing the risk of OHSS associated with higher doses of hCG supplementation in the luteal phase.

## Introduction

Over the past few decades, increasing knowledge of ovarian physiology and the possibility to evaluate accurately the ovarian reserve has led progressively to individualization and tailoring of ovarian stimulation for *in vitro* fertilization (IVF)—treatment.

Final oocyte maturation is the crucial step in ovarian stimulation cycles for IVF to retrieve mature oocytes for further processing in the IVF laboratory. In gonadotropin-releasing hormone (GnRH) antagonist cycles, human chorionic gonadotropin (hCG) and GnRH agonist can be administered for final oocyte maturation. Meanwhile, in patients with an expected or known high response, stimulation in a GnRH antagonist protocol with the use of GnRH agonist for final oocyte maturation is the state-of-the-art treatment to avoid or at least reduce significantly the risk for the development of ovarian hyperstimulation syndrome (OHSS) (1).

The GnRH agonist dislocates the GnRH antagonist in the pituitary from the GnRH receptors. This results in a surge of LH and FSH, inducing final oocyte maturation and ovulation (2). However, induced LH/FSH peak is shorter compared to a natural LH surge. The duration of the LH/FSH surge is critical for normal luteal function, and due to a shortened LH surge, the granulosa cells fail to complete luteinization, resulting in a corpus luteum with impaired secretory function and a shortened lifespan. Therefore, the luteal phase will be characterized by luteolysis and luteal phase insufficiency (3). Previously, it was assumed that all patients will develop severe luteolysis within a matter of 5 days after GnRH agonist trigger (3); however, recently, it was clearly demonstrated that luteolysis after GnRH agonist trigger is patient specific (4). Unfortunately, there are so far no established predictive parameters to estimate the severity of the luteolysis.

Furthermore, after introducing GnRH agonist for final oocyte maturation, the first studies using this approach reported a very poor reproductive outcome (5). Noticeably, standard luteal phase support with the use of vaginal progesterone could not counterbalance the severe luteal phase insufficiency, whereas the use of intensive luteal phase support, defined as daily 50 mg i.m. Progesterone and estradiol supplementation could lead to implantation, clinical, and ongoing pregnancy rates, which are comparable with the rates after hCG trigger (6).

Human chorionic gonadotropin attaches and activates the same receptor as LH, and in the absence of a LH signal from the pituitary, consequently hCG can rescue the corpus luteum function and prevent complete luteolysis, while it is administered within 72 h (7) in the appropriate dosage (8). Hence, several studies have shown that the administration of 1,500 IU hCG 35 h after GnRH agonist trigger result in comparable pregnancy rates in respect to hCG trigger. Nevertheless, still some patients will develop OHSS (9, 10).

A new approach in the treatment of OHSS high-risk-patients is the “luteal coasting” (11). This concept individualizes the luteal phase support after GnRH agonist administration for final oocyte maturation by monitoring the progesterone concentrations closely and administering a rescue dosage of hCG when progesterone concentrations drop significantly (12). However, heretofore, the hCG dosage and timing prerequisite to prevent luteolysis and sustain corpora lutea function and the progesterone levels required to achieve and maintain a pregnancy have not clearly been identified yet.

In this proof of concept study, we analyzed the correlation between progesterone levels 48 h after oocyte-pick-up (OPU) and hCG dosages administered as luteal phase support to search for the accurate amount of hCG for achieving a pregnancy and avoiding OHSS.

## Materials and Methods

### Patients

Information from 52 patients being treated between September 2015 and May 2017 for primary/secondary infertility and indication for ovarian stimulation for IVF/ICSI in IVI Middle East Fertility Clinic, Abu Dhabi, United Arab Emirates, and who received GnRH agonist for final oocyte maturation due to the risk for development of OHSS, as the ultrasound findings showed the existence of ≥13 follicles of a diameter ≥11 mm, as described by Papanikolaou et al. (13) was gathered for this retrospective study.

### Stimulation Protocol

Hormonal stimulation was performed in GnRH antagonist protocols with recombinant FSH (Puregon^®^, MSD; Gonal F^®^, MerckSerono) or human menopausal gonadotropin (Menogon^®^ or Menopur^®^, Ferring). The starting dosage was chosen according to the results of the anti-Müllerian hormone and antral follicle count (14). Starting on day 5, patients received a daily dosage of 0.25 mg GnRH antagonist (Orgalutran^®^, MSD or Cetrotide^®^, MERCK) to prevent premature ovulation.

During the stimulation course, stimulation dosage was adapted to the individual patient’s response. GnRH agonist trigger for final oocyte maturation was used to avoid OHSS as the ultrasound showed >13 follicles with a size of ≥11 mm (13). Patients received 0.3 mg of Triptorelin (Decapeptyl^®^, Ferring) for final oocyte maturation, as soon as ≥3 follicles were ≥17 mm in diameter. OPU was performed 36 h later under mild sedation, aspirating all follicles of a size of ≥11 mm.

### Luteal Phase Support

Patients started luteal phase support, using vaginal progesterone suppositories on the evening of the OPU day with 400 mg of progesterone (Cyclogest^®^, manufactured by Actavis, UK). From day OPU + 1, the dosage was increased to 3 × 400 mg.

Besides the luteal phase support through vaginal progesterone application, patients received individual dosages of hCG between 375 and 1,500 IU hCG 48 h after OPU. The administered hCG dosage did not follow a previously defined criteria; however, it was decided on the progesterone level 48 h after OPU and the clinical findings of the patient regarding abdominal swelling and lower abdominal pain. This approach was chosen for the high-responder patient as the fertilization law of the United Arab Emirates does not allow embryo freezing. The fertilization law of the United Arab Emirates (15) says in Article 11: “The Centre shall comply with the following with regard to the excess ova: … 2. In the case of surplus fertilized ova in whatever manner, these ova shall be left without medical attention until they perish naturally.”

### Endocrine Assessment

Serum endocrine assessment of progesterone (P4) 48 h after OPU procedure/84 h after final oocyte maturation was conducted to assess the pattern of individual luteolysis. The measurements of the progesterone level after OPU procedure were run as a clinical routine to evaluate the need of additional hCG administration for the luteal phase support.

Hormone levels (P4) were measured with the Cobas^®^ 6000 analyzer system, Roche. The upper measurable limit of progesterone levels with this system is 60 ng/ml; therefore, levels above this limit will be also expressed as 60 ng/ml.

For luteal phase support, additional to the vaginal progesterone application, hCG was administered after the result of progesterone measurement was available. The dosage was 375, 750, 1,000, or 1,500 IU hCG and was chosen depending on the progesterone level and the clinical condition of the patient in respect to abdominal swelling or lower abdominal pain, following the clinics’ established standard criteria and protocols and routine clinical practice.

Twelve days after embryo transfer (ET), a serum test for hCG was performed. The pregnancy test was considered to be positive when the hCG level was >15 IU/l. An “on going” pregnancy was defined once positive heartbeat was visible in the ultrasound at 12 weeks. The patients with a positive hCG test, however without visible ultrasonographic pregnancy sac, were counted as “pregnancy loss.” Patients with a negative pregnancy test were informed to stop the administration of vaginal progesterone.

The study was approved by the Ethic Committee of IVI Middle East Fertility Clinic, Abu Dhabi, United Arab Emirates (Research Ethics Committee IVI-ME_REC13_2017). Due to the fact that the herein analyzed measurements of progesterone levels were run as a clinical routine during IVF treatment, the ethic committee waived the need for obtaining oral or written approval from each patient.

### Data Analysis

Exploratory data analysis allowed the evaluation of data quality and detecting/correcting anomalies and was followed by a descriptive analysis and statistical summary of the data collected in the study. Categorical data are shown as proportions and 95% confidence intervals, while continuous variables are summarized using the mean, ranges, SD, and 95% confidence intervals.

Regarding the statistical analysis that was applied to fulfill each objective, to determine the association between quantitative values of the P4 48 h after the administration of GnRH agonist and the rate of pregnancy/on going pregnancy we initially checked the normal distribution by means of Kolmogorov–Smirnoff tests, and then Student’s *t*-test or Mann–Whitney’s test, depending on the normal distribution or not, of the main variable were applied.

To determine the association between the levels of progesterone 48 h after administration of GnRH agonist, the amount of hCG given 48 h after final oocyte maturation, and the rate of pregnancy/ongoing pregnancy, analysis was performed in the two subgroups: those patients with hCG ≤ 750 IU and the other one with hCG > 750 IU.

Data were analyzed with the Social Package for Social Sciences (SPSS) 23.0 software (Chicago, IL, USA), and statistical significance was established at *p* < 0.05.

## Results

The mean age of all 52 included patients was 30.75 years, the mean body mass index (BMI) of the patients was 26.33. 12 days after the ET, 35 patients (67.30%) had a positive pregnancy test and 17 patients (32.70%) had a negative result.

The mean ages of the patients with a positive pregnancy test and a negative pregnancy test were 30.40 and 31.47 years, respectively. The mean BMIs of the pregnant/the non-pregnant patients were 25.95 and 27.08, respectively.

In the whole group, the mean number of retrieved oocytes was 18.31 with a range from 9 to 31 oocytes. The mean number of oocytes was 18.54 oocytes for the patients with a positive pregnancy test and 17.82 for patients with a negative pregnancy test.

On the day of final oocyte maturation, the mean progesterone level for all patients was 0.73 ng/ml. For the patients with a positive pregnancy test and a negative pregnancy test, the mean progesterone levels were 0.70 and 0.78 ng/ml, respectively.

In all patients, the mean progesterone level 48 h after OPU was 29.89 ng/ml. Patients with a positive pregnancy test had the mean progesterone level of 28.97 ng/ml, and patients with a negative pregnancy test had the mean progesterone level of 31.79 ng/ml. There was no significant correlation between P4 level 48 h after OPU and the achievement of a pregnancy (*p* = 0.200).

The differences for the parameters age, BMI, number of retrieved oocytes, mean progesterone levels on the day of final oocyte maturation, and 48 h after OPU were statistically not significant.

The mean number of transferred embryos was 1.67 in the total group. A mean number of 1.86 embryos had been transferred in the pregnant patients and 1.29 embryos in the group of non-pregnant patients. The difference was statistically significant between the groups (*p* < 0.001).

Embryo transfer was performed on day 2 (1 case), day 4 (11 cases), day 5 (32 cases), and day 6 (8 cases). The results, including the ranges and the 95% confidence interval, are shown in Table 1.

**Table 1:** Summary of demographics and stimulation characteristics of the patients: all, pregnant, and non-pregnant patients.

Parameter	Mean	Range	95% confidence interval	Significance
**Age (years)**				
All patients	30.75	22–45	29.28–32.22	n.s.
Pregnant patients	30.4	22–43	28.68–32.12	
Non-pregnant patients	31.47	23–45	28.44–34.50	

**BMI (kg/m**^2^)				
All patients	26.33	18.15–36.6	25.21–27.44	n.s.
Pregnant patients	25.95	19.72–33.25	24.65–27.25	
Non-pregnant patients	27.08	18.15–36.16	24.78–29.37	

**Number of retrieved oocytes**				
All patients	18.31	9–31	16.85–19.76	n.s.
Pregnant patients	18.54	9–31	16.78–20.39	
Non-pregnant patients	17.82	9–29	14.96–20.69	

**Mean progesterone level day of final oocyte maturation (ng/ml)**				
All patients	0.73	0.24–1.38	0.66–0.80	n.s.
Pregnant patients	0.70	0.3–1.18	0.61–0.88	
Non-pregnant patients	0.78	0.6–1.2	0.69–0.88	

**Mean progesterone level 48 h after OPU (ng/ml)**				
All patients	29.89	13–56	27.06–32.73	n.s.
Pregnant patients	28.97	13–56	25.31–32.63	
Non-pregnant patients	31.79	18–45	27.09–36.48	

**Number of embryos transferred**				
All patients	1.67	1–2	1.54–1.8	*p* < 0.001
Pregnant patients	1.86	1–2	1.74–1.98	
Non-pregnant patients	1.29	1–2	1.05–1.54	

**Mean day of embryo transfer**				
All patients	4.81	2–6	4.62–4.99	n.s.
Pregnant patients	4.89	4–6	4.70–5.07	
Non-pregnant patients	4.65	2–6	4.20–5.09	

*Data expressed as means with their corresponding 95% CI*.

*BMI, body mass index; n.s., not significant; OPU, oocyte-pick-up*.

The further analysis evaluated the group of patients who achieved a pregnancy to investigate whether there is a correlation between progesterone levels 48 h after OPU and an ongoing pregnancy/pregnancy loss. Of 35 patients with a positive pregnancy test, 30 patients (85%) had an ongoing pregnancy and 5 patients (15%) lost the pregnancy before a viable pregnancy was seen in the ultrasound. Patients with an ongoing pregnancy had a mean P4 level of 29.98 ng/ml, and patients with a pregnancy loss had a mean P4 level of 22.90 ng/ml. The differences for the parameters age, BMI, number of retrieved oocytes, and mean progesterone levels on the day of final oocyte maturation and 48 h after OPU were statistically not significant. There was also no statistically significant difference in the number of transferred embryos in patients who had an ongoing pregnancy and those with a pregnancy loss (1.87 vs 1.80). There was no significant correlation between the P4 level 48 h after OPU and the outcome (on going pregnancy yes/no) of the pregnancy (*p* = 0.081). Table 2 summarizes the data of the subgroup analysis.

**Table 2:** Summary of subgroup analysis between patients with an ongoing pregnancy and a pregnancy loss.

	Ongoing pregnancy	Pregnancy loss	Significance (between the groups “ongoing pregnancy” and “pregnancy loss”)	Non-pregnant patients
Number of patients	30 (85%)	5 (15%)	n.a.	17 (32.7%)
Mean age (years)	30.80	28.00	n.s.	31.47
95% CI	28.85–32.75	24.38–31.62		28.44–34.50
BMI	26.27	24.08	n.s.	27.08
95% CI	24.78–27.75	22.06–26.11		24.78–29.37
Number of retrieved oocytes	18.73	17.40	n.s.	17.82
95% CI	16.75–20.72	12.46–22.34		14.96–20.69
Mean progesterone level day of final oocyte maturation (ng/ml)	0.72	0.58	n.s.	0.78
95% CI	0.62–0.83	0.29–0.88		0.69–0.88
Mean progesterone level 48 h after OPU (ng/ml)	29.98	22.90	n.s.	31.79
95% CI	26.24–33.72	5.97–39.82		27.09–36.48
Mean number of embryos transferred	1.87	1.80	n.s.	1.29
95% CI	1.74–2.00	1.24–1.36		1.05–1.54
Mean day of transfer	5	5	n.s.	4.88

*Data expressed as means with their corresponding 95% CI*.

*BMI, body mass index; n.a., not applicable; n.s., not significant; OPU, oocyte-pick-up*.

Human chorionic gonadotropin was given to the patient after the progesterone result was available to avoid luteal phase insufficiency. No patient developed early or late OHSS as a result of the hCG dosage administered for luteal phase support. 1 patient did not have any hCG at all during the luteal phase (progesterone level 40.0 ng/ml 48 h after OPU). hCG was administered in dosages of 375, 750, 1,000, or 1,500 IU to 12, 33, 4, and 2 patients, respectively. For those groups, the mean progesterone levels and the ranges were calculated. In the groups of hCG administration of 375, 750, 1,000, or 1,500 IU, mean progesterone levels were 37.03 ng/ml (range, 22–43 ng/ml), 28.26 ng/ml (range, 15.8–51.25 ng/ml), 20.9 ng/ml (range, 14–21.8 ng/ml), and 26.75 ng/ml, respectively.

To evaluate the influence of the dosage of administered hCG and the achievement of a pregnancy, correlation between hCG dosages above and below 750 IU hCG and the pregnancy outcome was performed.

Of the six patients receiving 1,000 or 1,500 IU hCG, four patients (66.66%) did not achieve a pregnancy and two patients (33.33%) had a positive pregnancy test. The mean progesterone level of the patients in this group was 22.87 ng/ml. The patients without positive pregnancy test had a mean P4 level of 27.55 ng/ml, and patients with a pregnancy had a mean progesterone level of 13.50 ng/ml. There was no significant correlation between the application of a hCG > 750 and the achievement of a pregnancy (*p* = 0.133).

46 patients received a dosage of 750 IU hCG or less. In this group, the mean P4 level was 30.81 ng/ml. Of this group, 13 patients (28.26%) had no pregnancy and 33 patients (71.73%) achieved a pregnancy. The mean P4 levels were 33.09 and 29.91 ng/ml, respectively. There was no significant correlation between the application of a hCG ≤ 750 and the achievement of a pregnancy (*p* = 0.2). Table 3 shows ranges, the 95% CI and the correlation between the applied hCG dosage and the achievement of a pregnancy.

**Table 3:** Correlation between hCG dosage >750 IU or ≤750 IU and achievement of pregnancy.

Amount of hCG for LPS	≤750 IU hCG		>750 IU hCG	
Number of patients	46		6	
Mean progesterone level and range 48 h after OPU (ng/ml)	30.81 (16–56)		22.87 (13–40)	
95% CI	27.87–33.74		11.75–33.99	

**Pregnancy**	**Yes**	**No**	**Yes**	**No**

Number of patients	33	13	2	4
Mean progesterone level 48 h after OPU (ng/ml)	29.91 (16–56)	33.09 (18–45)	13.50 (13–14)	27.55 (18–40)
95% CI	26.27–33.54	27.74–38.45	7.15–19.85	11.70–43.40
Correlation between pregnancy yes/no and hCG dosage	No (*p* = 0.2)		No (*p* = 0.131)	

*hCG, human chorionic gonadotropin; OPU, oocyte-pick-up*.

To evaluate a possible correlation in the pregnant patients between the amount of administered hCG (above 750 IU hCG or ≤750 IU hCG), a pregnancy, and the progesterone levels 48 h after OPU, those parameters were correlated with each other. Of the group of patients receiving 375 or 750 IU hCG, 33 patients had a positive pregnancy test. 29 of them (87.87%) had an ongoing pregnancy and four patients did not. In these groups, mean P4 levels were 25.12 ng/ml (SD, ±14.657) and 30.57 (SD, ±9.655), respectively. In patients with hCG administration of 375 or 750 IU, there was no significant correlation between P4 levels and an ongoing pregnancy (*p* = 0.184). Of the two patients, receiving previously 1,000 or 1,500 IU hCG, one patient had an ongoing pregnancy and the other patient had a biochemical pregnancy, with progesterone levels of 13 and 14 ng/ml, respectively. Table 4 shows the correlation between the applied hCG dosage, progesterone level 48 hours after OPU, and the ongoing pregnancy (yes/no).

**Table 4:** Correlation between hCG dosage >750 IU and ≤750 IU, progesterone level 48 h after OPU, and an ongoing pregnancy (yes/no).

Amount of hCG for LPS	≤750 IU hCG		>750 IU hCG	
Number of patients with pregnancy	33		2	

**Ongoing pregnancy**	**Yes**	**No**	**Yes**	**No**

Number of patients	29	4	1	1
Mean progesterone level 48 h after OPU (ng/ml)	25.12 (1.80–48.44)	30.57 (26.90–34.24)	13	14
Correlation between ongoing pregnancy and hCG dosage	No (*p* = 0.184)		n.s.	

*Data expressed as means with their corresponding 95% CI*.

*hCG, human chorionic gonadotropin; n.a., not applicable; n.s., not significant; OPU, oocyte-pick-up*.

## Discussion

To optimize the approach of “luteal coasting” and minimize the risk for development of OHSS after GnRH agonist trigger due to an inadequate high hCG dosage, knowledge of the interaction between progesterone levels in the early luteal phase and the “optimal” hCG dosage is extremely important.

After administration of GnRH agonist for final oocyte maturation, pulsatile LH secretion continues; however, the mean LH concentrations and LH pulse amplitudes are lower than those described for a natural cycle in the early luteal phase. Therefore, the process of luteolysis starts very early in the luteal phase, as progesterone and estradiol levels drop 2 days after ovulation (16). Maintenance of adequate levels of progesterone is crucial for implantation. The first studies, using a standard luteal phase support with vaginal progesterone after GnRH agonist trigger found a significantly lower implantation rate and clinical pregnancy rate in addition to a significantly higher rate of early pregnancy loss (5).

The optimal approach to maintain a sufficient luteal phase is still under debate notwithstanding the fact that the use of GnRH agonist for final oocyte maturation is nowadays the state-of-the-art treatment for expected or known high responder to avoid OHSS (9, 17, 18). This is especially important as the “freeze-all” and “cycle segmentation” policy (19) is not applicable for all patients due to various reasons.

Corpora lutea function can be easily maintained through the application of hCG by timely administration. The first studies administered an arbitrarily chosen bolus of 1,500 IU hCG 35 h after GnRH agonist trigger (9). Due to the occurrence of OHSS cases despite the reduced dosages (9, 10), other authors evaluated the administration of even lower hCG dosages some on a daily basis. However, even with these lower dosages, OHSS could not be avoided completely (20, 21). Therefore, further individualization of hCG administration in the context of the luteal coasting approach is required to reduce furthermore the OHSS risk after GnRH agonist trigger (12). The aim of this retrospective analysis is to evaluate the correlation between the applied hCG dosages, depending on the serum progesterone levels 48 h after OPU and the pregnancy rate as well as the ongoing pregnancy rate. The approach of administration of individualized hCG dosages, based on the individual luteolysis and the clinical findings of the patient was chosen, as the fertilization law of the United Arab Emirates does not allow embryo freezing.

This proof-of-concept-study supports the idea that early OHSS development seems to be avoidable by individualization of luteal phase support through the administration of reduced hCG dosages without impacting the ART outcome. However, it is very important that the hCG is administered timely enough to rescue the corpora lutea with the subsequent progesterone production. Those findings have to be supported by a bigger sample size.

The current analysis showed that achievement of a pregnancy as well as the outcome of the pregnancy [ongoing pregnancy (yes/no) until 12 weeks of gestation] are not correlated with the P4 levels we have mentioned, i.e., with 13 ng/ml being the lowest level in our study group 48 h after OPU (*p* = 0.200 and *p* = 0.0081). According to these results, it can be assumed that the amount of “progesterone drop-down” 48 h after OPU even toward a level of 13 ng/ml does not have any impact on the chance of achieving and/or maintaining a pregnancy, as long as a hCG bolus will rescue the corpora lutea function and therefore maintain a sufficient luteal phase support. However, the data regarding a lower limit of progesterone levels, which are still sufficient to achieve and maintain a pregnancy after the use of GnRH antagonist, are limited.

Moreover, no significant correlation between the application of a hCG bolus >750 or ≤750 and the achievement of a pregnancy (*p* = 0.133 and *p* = 0.2 respectively) was found. In addition, the amount of administered hCG does not seem to have an influence regarding the maintenance of the pregnancy, as there was no significant correlation between the P4 levels, the hCG dosage of ≤750 or >750 IU, and the outcome of the pregnancy [ongoing pregnancy (yes/no) until 12 weeks of gestation; *p* = 0.184 and *p* = 0.064 respectively].

These data indicate that the hCG dosage of 1,500 IU, previously chosen as a “rescue-bolus” (9) can be reduced on individual bases, without having a negative impact on the chance of pregnancy or the chance for an ongoing pregnancy.

Csapo et al. (22) demonstrated that early pregnancy can only be maintained under sufficient corpora lutea function or progesterone replacement in case of a loss of the corpus luteum. Luteal phase deficiency in a natural cycle is defined when the midluteal progesterone levels are measured below 10 ng/ml (31.8 nmol/l) or a sum of three random serum P measurements <30 ng/ml (95.4 nmol/l) (23). Hence, it is not clear whether these levels are also sufficient in an ART cycle due to the previous ovarian stimulation, which results in supraphysiological hormonal levels. Recently, it was demonstrated that in ART cycles, pregnancies can be achieved and maintained even with early luteal phase progesterone levels of 13 ng/ml when a hCG bolus was administered for luteal phase support (24).

All patients in this retrospective analysis administered 3 × 400 mg vaginal progesterone for luteal phase support, which will also contribute to the measured serum progesterone levels.

The contribution of the vaginal progesterone toward the serum progesterone levels cannot be determined. However, it can be assumed that the contribution was low as it was previously demonstrated that serum progesterone levels measured after vaginal progesterone administration are even lower than in natural cycle (25).

Our current data support the idea that a hCG dosage below 1,500 IU is sufficient to maintain adequate progesterone levels in early and mid-luteal phase and to allow implantation. From day 7 of fertilization, the embryo will start producing hCG by itself (26), and from that point of time, endogenous produced hCG will cover the LH deficit caused by the supraphysiologic steroid levels (27) and avoid regression of the corpora lutea.

The weakness of the current proof-of-concept study is the limited number of patients evaluated; therefore, statistical analysis of the outcomes was not feasible as the different embryo numbers transferred cannot be adjusted for, e.g., by logistical regression analysis. The hCG dosage did not follow previously defined criteria and was decided on the progesterone level 48 h after OPU and the clinical findings of the patient regarding abdominal swelling and lower abdominal pain. To define a treatment algorithm, future studies should evaluate the ART outcome in a prospective randomized controlled study with previously defined hCG dosage depending on the progesterone level.

## Conclusion

Luteal phase support after final oocyte maturation with GnRH agonist in a GnRH antagonist protocol is still under discussion. Most patients are receiving similar luteal phase supports and the administration of a hCG rescue bolus of 1,500 IU stills bears the risk of OHSS development. The current proof-of-concept study demonstrates that the hCG dosage can be individualized, and therefore, it seems that OHSS after GnRH agonist administration for final oocyte maturation can be avoided by dosage reduction of the hCG rescue bolus without impacting the chance of achieving and/or maintaining a pregnancy. Despite the small sample size, this proof-of-concept study is important to be seen as a possible approach in high-responder patients, in whom freezing of embryos might not be applicable due to various reasons.

## Ethics Statement

The retrospective analysis was approved by the Ethic Committee of IVI Middle East Fertility Clinic, Abu Dhabi, United Arab Emirates (Research Ethics Committee IVI-ME_REC13_2017).

## Author Contributions

BL: concepted the analysis and wrote the manuscript. SS: data collection. NG: statistical analysis of the data. LM: involved in patient’s treatment and review of manuscript. NE: review of manuscript. HF: supervision of the study concept and review of manuscript.

## Conflict of Interest Statement

The authors state that there is no conflict of interest. Also the research was conducted in the absence of any commercial or financial relationships.
